# Mouse models of surgical and neuropathic pain produce distinct functional alterations to prodynorphin expressing neurons in the prelimbic cortex

**DOI:** 10.1016/j.ynpai.2023.100121

**Published:** 2023-02-13

**Authors:** Shudi Zhou, Yuexi Yin, Patrick L. Sheets

**Affiliations:** a Medical Neurosciences Graduate Program, Indiana University School of Medicine, Indianapolis, IN 46202, USA; b Stark Neurosciences Research Institute, Indiana University School of Medicine, Indianapolis, IN 46202, USA; c Department of Pharmacology and Toxicology, Indiana University School of Medicine, Indianapolis, IN 46202, USA

**Keywords:** Pain models, Prodynorphin, Electrophysiology, Brain slice, Prelimbic cortex, Sex differences

## Abstract

The medial prefrontal cortex (mPFC) consists of a heterogeneous population of neurons that respond to painful stimuli, and our understanding of how different pain models alter these specific mPFC cell types remains incomplete. A distinct subpopulation of mPFC neurons express prodynorphin (Pdyn^+^), the endogenous peptide agonist for kappa opioid receptors (KORs). Here, we used whole cell patch clamp for studying excitability changes to Pdyn expressing neurons in the prelimbic region of the mPFC (PL^Pdyn+^ neurons) in mouse models of surgical and neuropathic pain. Our recordings revealed that PL^Pdyn+^ neurons consist of both pyramidal and inhibitory cell types. We find that the plantar incision model (PIM) of surgical pain increases intrinsic excitability only in pyramidal PL^Pdyn+^ neurons one day after incision. Following recovery from incision, excitability of pyramidal PL^Pdyn+^ neurons did not differ between male PIM and sham mice, but was decreased in PIM female mice. Moreover, the excitability of inhibitory PL^Pdyn+^ neurons was increased in male PIM mice, but was with no difference between female sham and PIM mice. In the spared nerve injury model (SNI), pyramidal PL^Pdyn+^ neurons were hyperexcitable at both 3 days and 14 days after SNI. However, inhibitory PL^Pdyn+^ neurons were hypoexcitable at 3 days but hyperexcitable at 14 days after SNI. Our findings suggest different subtypes of PL^Pdyn+^ neurons manifest distinct alterations in the development of different pain modalities and are regulated by surgical pain in a sex-specific manner. Our study provides information on a specific neuronal population that is affected by surgical and neuropathic pain.

## Highlights

• A subset of neurons in the prelimbic cortex (PL) express prodynorphin (Pdyn).
• PL^Pdyn+^ neurons consist of excitatory and inhibitory subtypes.
• Paw incision induces transient hyperexcitability of excitatory PL^Pdyn+^ neurons.
• Nerve injury induces sustained hyperexcitability of excitatory PL^Pdyn+^ neurons.

## 1 Introduction

Neurons in the medial prefrontal cortex (mPFC) respond to noxious (i.e. painful) stimuli (Condes-Lara et al., 1989) and play an important role in the emotional valence, attentional components and the descending modulation of pain (Porro et al., 2002, Lee et al., 2015, Martinez et al., 2017, Dale et al., 2018, Zhou et al., 2018). The mPFC is comprised of a heterogeneous population of glutamatergic (i.e. excitatory) pyramidal neurons and GABAergic (i.e. inhibitory) neurons that receive, integrate, and relay synaptic information coming from both intracortical (local) and subcortical (long range) origins. Over a decade of work confirms epigenetic, morphological, functional, and circuit changes to mPFC neurons in multiple rodent models of pain (Cao et al., 2009, Metz et al., 2009, Alvarado et al., 2013, Blom et al., 2014, Alvarado et al., 2015, Cordeiro Matos et al., 2015, Zhang et al., 2015, Kelly et al., 2016, Kiritoshi et al., 2016, Cheriyan and Sheets, 2018, Shiers et al., 2018, Huang et al., 2019, Mitric et al., 2019, Cheriyan and Sheets, 2020, Jones and Sheets, 2020). However, these reported changes vary across distinct neuronal subtypes, laminar locations and substructures within the mPFC (Cheriyan and Sheets, 2018, Mitric et al., 2019, Jones and Sheets, 2020, Jefferson et al., 2021). This variability indicates that different forms of pain engage specific neuronal populations across anatomically distinct mPFC circuits.

One method for categorizing cortical neurons is by the expression of distinct neurotransmitters, which can provide insight into their function. The opioid neuropeptide dynorphin (Dyn) is the endogenous ligand for the kappa opioid receptor (KOR) (Chavkin et al., 1982), which is a receptor shown to be involved in mediating the negative affective component of pain. Prodynorphin (Pdyn), the precursor peptide to Dyn, is expressed in the prefrontal cortices of both humans and non-human primates (Khachaturian et al., 1985, Peckys and Hurd, 2001). In mice, a subset of cortical neurons express Pdyn and are mainly located in superficial laminar layers (i.e., laminar 2) including the mPFC (Pina et al., 2020). Increased expression of Pdyn mRNA is detected in the mPFC in both rat and mouse chronic pain models (Candeletti and Ferri, 1995, Palmisano et al., 2018), but is unchanged early (POD 4) after surgical incision (Nwaneshiudu et al., 2019). This indicates that chronic pain may be altering the activity of Pdyn expressing neurons in the mPFC (mPFC^Pdyn+^ neurons). However, functional changes to mPFC^Pdyn+^ neurons in surgical and chronic pain models yet to be identified. In this study, we used whole cell patch clamp electrophysiology in acute brain slices to study how the activity of Pdyn expressing neurons in the prelimbic cortex (PL^Pdyn+^ neurons), a subregion of mPFC, changes in mouse models of both postoperative pain and neuropathic pain.

## 2 Material and methods

### 2.1 Ethical approval and responsible use of animals

The Institutional Animal Use and Care Committee (IACUC) of the Indiana University School of Medicine approved (protocol# 19144) all procedures and experiments presented in this study. Experimenters maximized efforts to reduce animal use and animal suffering. Unfortunately, alternatives to ex-vivo and in-vivo techniques were not feasible for this project.

### 2.2 Animals

To obtain transgenic mice expressing the red fluorescent protein TdTomato specifically in neurons that share the neuronal lineage marker prodynorphin (Pdyn), female Pdyn-IRES-Cre (B6;129S-^Pdyntm1.1(cre)Mjkr^/LowIJ; stock #027958; Jackson labs) mice were mated with male Ai14 (B6;129S6-Gt(ROSA)^26SORtm14(CAG-tdTomato)Hze^/J; stock #007914; Jackson Labs) mice. All experiments were conducted in Pdyn TdTomato offspring of both sexes in accordance with the animal care and use guidelines of Indiana University, the National Institutes of Health, and the Society for Neuroscience. All mice were housed in a temperature controlled (21 ± 2 °C) vivarium with a 12 h/12 h light/dark cycle (lights on at 7 AM). Mice (total n = 106) of the same sex were housed together before PIM, SNI or sham surgeries with unrestricted access to food and water. Animals were randomly assigned to each group. After surgery, mice were single housed. Experiments were performed when mice reached a minimum age of 8 weeks. Proper pain controls were conducted to minimize animal suffering.

### 2.3 Intracranial virus injection

For intracranial injection, mice (postnatal day 42–49) were anesthetized with 1.5 % isoflurane in 100 % O_2_ at a flow rate of 1.0 L/min (SurgiVet Isotech 4, Smith). Artificial tears ointment (Rugby) was applied to the eyes after induction of anesthesia and a feedback controlled heating pad (FHC; Bowdoin, ME) connected with a thermometer was used to maintain body temperature at 37 °C. Mouse head was placed in a stereotaxic apparatus (900 series, Kopf Instruments). The top of the mouse head was shaved and was aseptically prepared by using 3 skin preparation protocols: povidone iodine (7.5 %, Purdue Products LP) and 70 % isopropyl alcohol wipes (Curity). The skull was exposed by incising the scalp using a No.11 blade (Royal-tek), and a Ram Power hand drill (MHC) was used to make a craniotomy for the placement of the injection needle. The coordinates for PL injection were (relative to bregma; in mm): 0.2 lateral, 1.7 rostral and 0.7 deep. Single injections (70 nL/injection; 75nL/min) of AAV1-EF1a-DIO-hChR2(E123A)-EYFP (Addgene, #35507-AAV1) were targeted to right PL using the UltraMicroPump controlling a Gastight 1701 Hamilton syringe paired with a beveled, 2 in., 27 gauge removable needle. After each injection, the Hamilton syringe was left in place for 7 min to prevent backflow before being removed slowly. For local anesthesia, lidocaine hydrochloride jelly USP (2 %, Akorn) was applied to the scalp by sterile cotton swabs before closing the incision with VetBond (3 M, St. Paul, MN). Subcutaneous injection of 5 mg/Kg Meloxicam (0.06 mg/Kg, Norbook) and buprenorphine hydrochloride (0.3 mg/mL 0.06 mg/Kg, PPR, NY) were applied to prevent inflammation and pain. Mice were monitored every day after the injection by a veterinarian from Laboratory Animal Research Center, Indiana University, School of Medicine, and were allowed to recover from the injection for at least 4 days before neuropathic pain model.

### 2.4 Plantar incision model (PIM) of postoperative pain

Mice (≥8 weeks) were briefly (5–10 s) anesthetized with 1.5 % isoflurane in 100 % O_2_ at a flow rate of 1.0 L/min (SurgiVet Isotech 4). Anesthetized mice were placed on bite bar in a rebreathing anesthetic circuit with nose cone (Vetamac, Rossville, IN). Artificial tears ointment (Dechra, UK) was applied after induction of anesthesia and a feedback controlled heating pad (FHC) was used to maintain body temperature at 37 °C. The plantar surface of the left hind paw was aseptically prepared by using three skin preparation protocols: povidone iodine (7.5 %, Purdue Products LP) and 70 % isopropyl alcohol wipes (Curity). We implemented the plantar Incision model (PIM) adapted from previously described methods (Pogatzki and Raja, 2003, Cowie and Stucky, 2019). After aseptic preparation, a 5 mm longitudinal Incision was made using a No.11 scalpel blade through the skin and fascia on the plantar surface of the left hind paw, starting 2 mm from the proximal end of the heel and extending toward the toes. The underlying plantaris muscle was elevated using pointed tips tweezers, leaving the muscle intact. A 5 mm longitudinal Incision was made along the center of the exposed plantaris muscle. The skin Incision was closed with two horizontal simple interrupted sutures of 5–0 nylon (McKesson) on the proximal and distal end of the Incision covered with triple antibiotic ointment (Actvis Pharma, Inc). Mice were allowed to recover from anesthesia in their home cages with wet feed and on a heating pad (FHC) before being returned to the vivarium. Sham mice underwent anesthesia, antiseptic preparation and topical triple antibiotic ointment application, but without Incisions on the skin, fascia, or the muscles. Mice were checked every day, and any mouse with evidence of infection or dehiscence, or lose of sutures were excluded from the study. For pain recovery studies, nylon sutures were removed when mice were anesthetized after postoperative day 1 (POD1) behavior measurement.

### 2.5 Spared nerve injury (SNI) model of neuropathic pain

For each group, the experimenter was blinded to the baseline von Frey results and randomly selected one mouse to be SNI and one to be sham. Mice were weighed and briefly anesthetized in an anesthesia box with 1.5–2.5 % isoflurane in 100 % O_2_ at a flow rate of 0.8–1.0 L/min. The snout of the mouse was then placed into a flexible nose cone connected to the isoflurane vaporizer allowing for continued anesthesia. Body temperature was maintained at 37 **°**C using a feedback controlled heating pad. The lateral surface of the left hind leg was shaved and disinfected using betadine and isopropyl alcohol. An approximately 4 mm incision through the skin was made and the underlying muscle layers were separated by blunt dissection using saline moistened sterile wooden dowels. The trifurcation of the left sciatic nerve was visualized. For SNI mice, an approximately 2 mm section of the tibial and common peroneal nerves distal to the trifurcation was removed, leaving the sural nerve intact. For sham mice, the trifurcation was exposed and visualized but not manipulated. The muscle layers of both SNI and sham mice were replaced and the outer skin layers were glued together using Vetbond. All mice recovered in a clean home cage with *ad libitum* water and wet feed on a heating pad for at least 30 min before being returned to the vivarium. Mice were monitored for 4 days post operation for signs of excessive pain such as reduced eating, drinking, activity, or grooming.

### 2.6 Assessment of sensory pain behavior

The behavior testing apparatus was an elevated wire mesh platform fabricated by Sheets lab personnel. Mice were acclimated separately inside 6 in. tall, 3 in. diameter Plexiglas tubes for 1 h before baseline testing. On testing day, standard von Frey filaments were used for (Touch Test, VWR) the standard up-down (SUDO) method (Chaplan et al., 1994, Bonin et al., 2014) to assess the mechanical allodynia via 50 % paw withdrawal threshold (PWT). The baseline PWT was acquired the day before the plantar incision. For PIM mice, Von Frey filaments were presented to the area between the two sutures, 1 mm medial to the incision. The filament was applied perpendicular to the plantar aspect of the hind paw until it was slightly bent for approximately 5 s. Filaments were presented at intervals of at least 30 s. Positive responses were considered as sharp withdrawal of the hind paw, flinching, and licking upon the application of the filament. The test was initiated using the 2.00 g filament. If no response was observed, the next stiffer filament was applied until a positive response was evoked; however, if a positive response was observed, a less stiff fiber was applied. The test was terminated four trials after the first positive response per the SUDO method. For the pain recovery studies, von Frey testing was performed at POD1, POD4, and POD7. Starting from POD7, von Frey testing was performed daily until pain behavior for each mouse returned to baseline for two consecutive days. For SNI studies, pain assessments were performed at baseline (before SNI or sham surgery) and POD3 or POD14. Von Frey filaments were presented to the area innervated by the sural nerve of both hind paws and were measured via von Frey filaments to evaluate the development of mechanical allodynia. The experimenter was blinded to the surgical group of the mice throughout the experiment.

### 2.7 Acute brain slice preparation

After the final behavioral test needed for a particular pain (i.e. PIM or SNI) or control group (i.e. sham), acute brain slices were prepared as described previously. Mice were briefly (∼15 s) anesthetized with 99.9 % isoflurane (Patterson Veterinary) and quickly decapitated. Brains were rapidly dissected and submerged in an ice cold choline solution containing the following (in mM): 110 choline chloride, 25 NaHCO_3_, 25 d-glucose, 11.6 sodium ascorbate, 7 MgSO_4_, 3.1 sodium pyruvate, 2.5 KCL, 1.25 NaH_2_PO_4_, and 0.5 CaCl_2_). Coronal brain slices containing mPFC (spine of the blade tilted rostrally 15° off vertical plane, 300 µm thick) were prepared with a vibratome (VT1200S; Leica). Slices were transferred to a 37 °C artificial CSF (ACSF) bath containing the following (in mM): 127 NaCl, 25 NaHCO_3_, 25 d-glucose, 2.5 KCl, 1MgCl_2_, 2 CaCl_2_, and NaH_2_PO_4_ for 30 min. Slices were subsequently incubated for at least 45 min in ACSF at room temperature before being transferred to the recording chamber.

### 2.8 Electrophysiological recordings

Slices were placed in the recording chamber of a SliceScope Pro 6000 (Scientifica) and continuously perfused with the ACSF (30–32 °C) at the rate of ∼ 1 mL per minute. Slices were held in place with a slice anchor (Warner Instruments). Fluorescently labeled PL^Pdyn+^ neurons were identified with a LED illumination system (CoolLED pE-4000) using a 580 nm wavelength LED with a RFP filter (ET FITC/RFP, Olympus). Recording pipets were made from borosilicate capillaries with filaments (G150-F; Warner Instruments) using a horizontal pipet puller (P-97; Sutter Instruments). PL^Pdyn+^ neurons approximately 60 µm or deeper from the surface of the slice were targeted for recording. After establishing a Gigaohm (GΩ) seal between the pipette tip and the cell membrane gentle negative pressure was applied from inside the pipette to open the cell membrane. After the opening of the cell membrane, neurons were allowed to stabilize for 5 min before recording. For intrinsic whole cell recordings, the patch electrode (3–4 MΩ resistance) were filled with (in mM) 128 K-gluconate, 10 HEPES, 1 EGTA, 4 MgCl_2_, 4 ATP, 0.4 GTP, 10 phosphocreatine, 3 ascorbate plus 3.5 mg/ml biocytin (Sigma-Aldrich). Whole cell patch clamp recordings were performed at 30–32 °C, amplified and filtered at 4 kHz, and digitized at 10 kHz using a Multiclamp 700B amplifier (Molecular Devices). Membrane potential was held at −70 mV in a voltage clamp mode. No synaptic blockers were added during intrinsic electrophysiological recordings. In *ex vivo* optogenetics, CPP and NBQX (Tocris, Bristol, UK; 5 μM) were added to the ACSF to block glutamatergic (excitatory) inputs; SR 95531 hydrobromide (GABAzine, Tocris, Bristol, UK; 10 μM) was added to the ACSF to block GABAergic (inhibitory) inputs. Pipette capacitance was compensated and the inclusion of data required a series resistance < 35 MΩ. Current clamp recordings were bridge balanced. The experimenter was blinded to the surgical group of the mice throughout the recording.

### 2.9 Morphological identification of neurons following electrophysiological recordings

After each recording, the patch pipette was slowly withdrawn to allow the cell membrane to reseal. Slices containing successfully resealed neurons were transferred back to the incubating chamber containing ACSF to allow for adequate filling of soma and dendrites with biocytin. Slices were first fixed in 4 % paraformaldehyde/phosphate buffered solution (PFA/PB) overnight, and then transferred to PB. Fixed brain slices were first washed 3 × 10 min in 0.1 % PBST individually before being placed in 1 mL blocking solution containing 0.3 % PBST and 5 % normal goat serum (EMD Millipore Corp, USA) on a rotator for 1 h at room temperature. Then slices were incubated with 1:1000 streptavidin, Alexa Fluor 488 conjugated green fluorescent dye (Invitrogen) for visualizing dendritic morphology for 1 h. Slices were then washed 3 × 10 min in 0.1 % PBST. After rinsing, samples were placed on cover slips and mounted on microscope slides with ProLongTM Gold antifade mountant (Invitrogen, United States). Brain sections were protected from light at every step.

### 2.10 Tissue preparation and immunohistochemistry

Mice were anesthetized by intraperitoneal administration of a mixture of ketamine (87.5 mg/kg, Henry Schein, Dublin, OH) and xylazine (12.5 mg/kg, Akorn Animal Health, Lake Forest, IL). Then mice were perfused with 1X PBS followed by 4 % PFA/PBS. Brains were removed and post fixed in 4 % PFA/PBS for 24 h before being transferred to 30 % sucrose at 4 °C. Coronal sections (50 µm) were cut in the rostral to caudal direction using a vibratome (VT1200S; Leica). Free floating sections were washed in 0.1 % PBST (3 × 10 min) individually before being blocked with 1 mL block solution on a rotator for 1 h at room temperature. Then brain sections were washed in 0.1 % PBST (3 × 10 min). Slices were first incubated with primary antibody (chicken GAD1/67, Synaptic System, Germany, 1:500; rabbit Pdyn polyclonal antibody, ThermoFisher, PA5-22286, 1:500) in block solution overnight at 4 °C, then washed in 0.1 % PBST (3 × 10 min). Brain sections were next incubated with secondary antibody (Alexa FluorTM 488 goat anti-chicken IgG, 1:500; Alexa FluorTM 488 goat anti-rabbit IgG, 1:500, Invitrogen, United States) with block solution for 2 h at room temperature. After rinsing, slices were placed on cover slips and mounted on microscope slides with Fluoromount-GTM, with DAPI (Invitrogen, United States). Brain sections were protected from light at every step. Fluorescent images were captured by using All-in-One Fluorescence Microscope, BZ-X800 (Keyence, Itasca, IL) using 20 X air objective.

### 2.11 Quantification and statistical analysis

Analysis of recording data was performed offline using Custom MATLAB (The MathWorks, RRID:SCR_001622). All of the statistical details of the experiments can be found in the figure legends. Behavior data for Sham/PIM POD 1 and sham/SNI were analyzed by ordinary-two-way ANOVA. Behavior data for Sham/PIM recovery were analyzed by repeated measurement (RM) two-way ANOVA (GraphPad Prism 8). For the intrinsic data comparison, unpaired Student’s *t*-test was used for normally distributed data, while Wilcoxon rank-sum test is used for non-parametric data. Differences were considered significant a p < 0.05. For excitability comparison, data were analyzed by repeated measurement two-way ANOVA (GraphPad Prism 8). Unless otherwise noted, results are presented as mean ± SD. For data presented as boxplots, the box displays the central 50 % of the data with the central line indicating the median and the lower/upper boundary lines being the 25 %/75 % quantile of the data. The outliers are plotted individually using the '+' marker symbol. Cell counting for IHC was conducted manually.

## 3 Results

### 3.1 Prodynorphin expressing neurons in the prelimbic cortex are heterogeneous

**Fig. 1:**
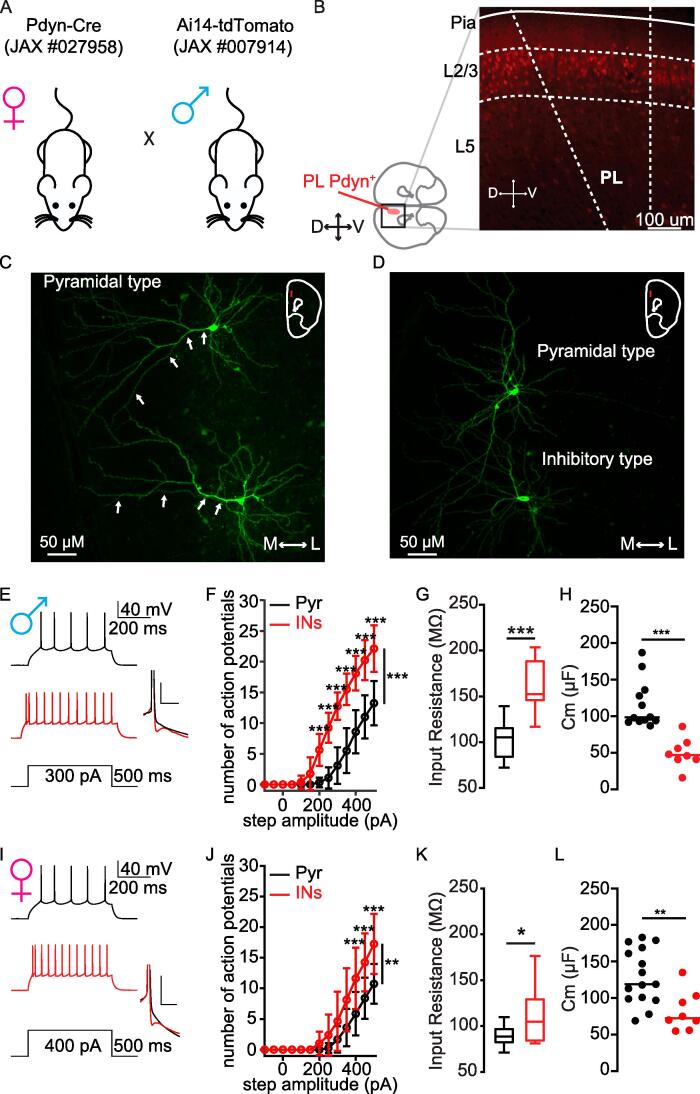
**Prodynorphin-expressing neurons in the prelimbic cortex are heterogeneous. A.** Schematic showing the generation of Pdyn-cre-tdTomato mice by crossing Pdyn-cre females with Ai14-tdTomato reporter males. **B.** Representative confocal image (10 X) of a coronal slice showing that Pdyn^+^ neurons are predominately expressed at L2 of the prelimbic (PL) cortex (bottom left: orientation of the brain slice and location of the PL cortex; D = dorsal; V = ventral). **C.** Representative confocal image (20 X) of pyramidal PL^Pdyn+^ neurons with typical apical dendrite structure (white arrows). **D.** Representative confocal image (20 X) of an inhibitory PL^Pdyn+^ neuron (lower) and a pyramidal PL^Pdyn+^ neuron (upper). **E-L.** Electrophysiological comparison of pyramidal (black) and inhibitory (red) L2 PL^Pdyn+^ neurons recorded from male (**E-H**) and female (**I-L**) mice. **E.** Representative traces showing action potentials of a pyramidal PL^Pdyn+^ neuron (black) and an inhibitory PL^Pdyn+^ neuron (red) from sham POD1 male mice. Inset, afterhyperpolarization comparison between pyramidal and inhibitory PL^Pdyn+^ neuron. (scale bar, horizontal 100 ms, vertical 100 mV). **F.** The relationship of AP firing with current step amplitude from pyramidal (n = 12 from 9 slices in 5 mice) and inhibitory PL^Pdyn+^ neuron (n = 8 from 4 slices in 3 mice) in sham POD1 male mice (RM two-way ANOVA, F_surgery_ (1, 18) = 58.83, *** P < 0.001, F_current_ (12,216) = 252.8, P < 0.001, and F_interaction_ (12,216) = 29.42, *** P < 0.001; Bonferroni post hoc, *** P < 0.001). Error bars = S.E.M.. **G.** Pyramidal and inhibitory PL^Pdyn+^ neurons are significant different in input resistance in sham POD1 male mice (t(18) = -5.507, *** P < 0.001; Error bars = S.E.M.; Student’s *t*-test). **H**. Pyramidal PL^Pdyn+^ neurons have significant higher membrane capacitance than inhibitory PL^Pdyn+^ neurons in sham POD1 male mice (t(18) = 5.09, *** P < 0.001; Student’s *t*-test). **I.** Representative traces showing action potentials of a pyramidal PL^Pdyn+^ neuron (black) and an inhibitory PL^Pdyn+^ neuron (red) from sham POD1 female mice. Inset, afterhyperpolarization comparison between pyramidal and inhibitory PL^Pdyn+^ neuron. (scale bar, horizontal 100 ms, vertical 100 mV). **J.** The relationship of AP firing with current step amplitude from pyramidal (n = 15 from 10 slices in 6 mice) and inhibitory PL^Pdyn+^ neuron (n = 8 from 6 slices in 4 mice) in sham POD1 female mice (RM two-way ANOVA, F_surgery_ (1, 21) = 9.936, ** P = 0.005, F_current_ (12,252) = 136.3, *** P < 0.001, and F_interaction_ (12,252) = 9.470, *** P < 0.001; Bonferroni post hoc, *** P < 0.001). Error bars = S.E.M. **K.** Pyramidal and inhibitory PL^Pdyn3+^ neurons are significant different in input resistance in sham POD1 female mice (t(21) = -2.2928, * P = 0.03; Error bars = S.E.M.; Student’s *t*-test). **L.** Pyramidal PL^Pdyn+^ neurons have significant higher membrane capacitance than inhibitory PL^Pdyn+^ neurons in sham POD1 female mice (t(21) = 3.13, ** P = 0.005; Student’s *t*-test). (For interpretation of the references to colour in this figure legend, the reader is referred to the web version of this article.)

We genetically labeled neurons that share the neuronal lineage marker prodynorphin (Pdyn) with the red fluorescent protein tdTomato by crossing Pdyn-Cre female mice with Ai14 floxed tdTomato reporter mice **(**Fig. 1**A; see Methods)**. Confocal imaging of coronal slices showed that Pdyn^+^ tdTomato neurons are extensively expressed in laminae 2/3 (L2/3) of the prelimbic (PL) cortex, a subregion with the mPFC, with sparser expression at deeper laminar layers **(**Fig. 1**B)**. We used confocal microscopy to examine the morphology of L2 PL^Pdyn+^ neurons following whole cell electrophysiological recordings. We found a majority of L2 PL^Pdyn+^ neurons displayed apical dendrites characteristic of pyramidal (glutamatergic) neurons **(**Fig. 1**C)**, while a minority displayed a smaller soma and lacked a clear apical dendrite indicative of inhibitory (GABAergic) neurons **(**Fig. 1**D)**. Analysis of recording data showed that L2 PL^Pdyn+^ neurons with a clear apical dendrite displayed both lower firing rates and smaller input resistance compared to those neurons lacking an apical dendrite **(**Fig. 1**E-L,** Table 1**)**. L2 PL^Pdyn+^ neurons without a clear apical dendrite exhibit significant fast afterhyperpolarization **(**Fig. 1**E, I, insets)**. Most notable was that pyramidal L2 PL^Pdyn+^ neurons displayed a significantly larger membrane capacitance **(**Fig. 1**H, L)**, which correlates with larger size. Neurons with uncertain morphology or poor imaging were excluded from electrophysiological analyses. Combining data from the morphological and electrophysiological analyses for both male and female mice, 186 (55 %) of recorded L2 PL^Pdyn+^ neurons in this study were pyramidal neurons, while 151 (45 %) were inhibitory neurons**.** We recognize that this recording data is not consistent with our IHC data (24 % PL^Pdyn+^ neurons express GAD1/GAD67). This discrepancy is likely due to recorded neurons that could not be morphologically identified being excluded from analyses.

**Table 1:** Comparison of pyramidal and inhibitory PL^Pdyn+^ neurons from male and female sham mice, related to Fig. 1.

**PL^Pdyn+^ neurons**	**Male**		**Female**	
	PYR (n = 12 neurons; 5 mice)	INs (n = 9 neurons; 4 mice)	PYR (n = 15 neurons; 6 mice)	INs (n = 8 neurons; 4 mice)
**Subthreshold properties**				
Resting potential (mV)	−86.3 ± 3.81	−78.1155 ± 9.4809 ^#*^	−87.7 ± 3.93	−86.7 ± 2.57
Input resistance (mΩ)	100 ± 20.42	156 ± 32^#***^	89.8 ± 3.1	111 ± 11.8^#*^
**Firing properties**				
Threshold (mV)	−39.6 ± 3.19	−37.127 ± 4.48	39.4 ± 3.51	−40.1 ± 4.53
Threshold (pA)	300 (1 0 0)^⊺^	200 (50)^⊺$***^	350 (50)^⊺^	275 (1 2 5)^⊺^
Frequency/current (Hz/pA)	0.1 ± 0.026	0.14 ± 0.021^$**^	0.1 ± 0.016	0.13 ± 0.021^#***^
APs @400 pA	8.5 ± 3.53	17.44 ± 3.32^#***^	8.4 ± 3.36	14.25 ± 4.74
Height (mV)	73.5 ± 8.8	63.73 ± 15.08^#*^	73 ± 9.03	74 ± 10.45

#: Student’s unpaired *t*-test; Data shown as mean ± standard deviation.

$: Mann-Whitney *U* test; Data shown are median ± standard deviation.

*: p < 0.05; **: p < 0.01; ***: p < 0.001.

⊺: median (interquartile range).

**Fig. 2:**
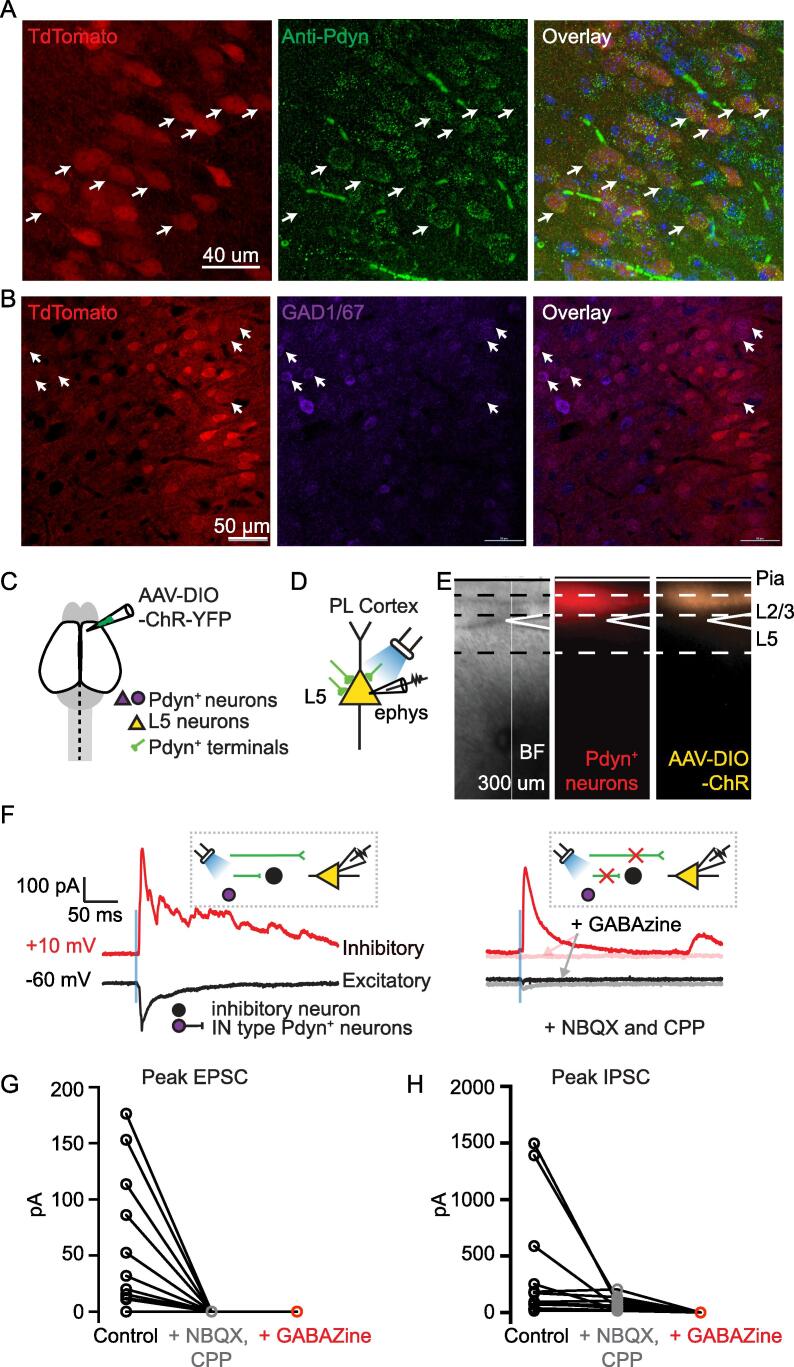
**PL^Pdyn+^ neurons send local excitatory and inhibitory inputs to layer 5 (L5) cortical neurons. A.** Confocal images showing Pdyn tdtomato neurons (left), anti-Pdyn (middle), and overlay (right). Quantification from 3 Pdyn tdTomato male mice, 3 slices in totals. **B.** Confocal images showing Pdyn tdtomato neurons (left), GAD1/67 (middle), and overlay (right). Quantification from 3 Pdyn tdTomato male mice, 3 slices in totals. **C.** Injection of AAV-DIO-ChR2-YFP into the PL of Pdyn-cre-tdTomato mice. **D.** Schematic showing electrophysiological recording from a L5 unlabeled cortical neuron in PL cortex while photoactivating ChR2 labeled Pdyn^+^ neuronal terminals. **E.** Representative coronal brain sections (Left: bright field; Middle: 580 nm; Right: 470 nm). **F.** Examples of evoked EPSC (black trace; holding at −60 mV) and evoked IPSC (red trace; holding at + 10 mV) recorded during wide-field activation of ChR2 Pdyn^+^ terminals via blue LED stimulation before (left) and after (right) adding synaptic blockers (CPP and NBQX). **Left inset**, schematic showing potential sources of local synaptic input to L5 PL unlabeled cortical neurons during photoactivation. **Right,** examples of evoked EPSC (black trace; holding at −60 mV) and evoked IPSC (red trace; holding at + 10 mV) recorded in the presence of glutamatergic blockers (solid black + red, CPP 5 µM, NBQX 5 µM) and GABAergic blockers (transparent black + red, GABAzine 10 µM). **Right inset**, schematic showing potential synaptic inputs to L5 PL cortical neurons during photoactivation while blocking excitatory and inhibitory connections. **G, H**. Excitatory (**G**) and inhibitory (**H**) input values from L2/3 Pdyn^+^ neurons to L5 neurons following treatment with glutamatergic blockers (CPP 5 µM, NBQX 5 µM) and GABAergic blockers (GABAzine 10 µM). (For interpretation of the references to colour in this figure legend, the reader is referred to the web version of this article.)

It has been shown that Pdyn expression changes during rodent development (Alvarez-Bolado et al., 1990). We therefore stained a subset of brain slices from Pdyn^+^ tdTomato mice with an anti-Pdyn antibody. We find that approximately 84.3 % of the neurons labeled with tdTomato in the PL cortex are co-labeled with Pdyn antibody **(**Fig. 2**A)**. Based on this finding, we cannot exclude the possibility that a small percentage of Pdyn TdTomato neurons recorded were not actively expressing Pdyn. Previous studies indicate that Pdyn^+^ neurons in the cortex are heterogeneous, consisting of both excitatory pyramidal and inhibitory neurons (Sohn et al., 2014, Loh et al., 2017, Smith et al., 2019). Immunohistochemistry showed that 24 % of the PL^Pdyn+^ neurons express glutamate decarboxylase 1 (GAD1/GAD67), which is a molecular marker for GABAergic neurons **(**Fig. 2**B)**. To test the nature of local synaptic outputs from PL^Pdyn+^ neurons, we injected AAV1-EF1a-DIO-hChR2(E123A)-EYFP into the PL cortex of Pdyn-Cre-tdTomato mice **(**Fig. 2**C),** allowing for optogenetic control of PL^Pdyn+^ neurons. Given the difficulty of isolating AAV infection in only L2, these experiments consisted of testing local connections from PL^Pdyn+^ neurons in both L2 and L3 (L2/3). After adequate time for hChR2(E123A) expression in L2/3 PL^Pdyn+^ neurons (∼14 days), we prepared acute coronal brain slices and recorded from unlabeled L5 neurons in the PL cortex **(**Fig. 2**D, E)**. We detected both excitatory (inward) and inhibitory (outward) currents following broad field optogenetic activation of hChR2(E123A)-expressing PL^Pdyn+^ neurons **(**Fig. 2**F-H)**. We found that a small subpopulation of L5 cortical neurons only receive inhibitory inputs from L2/3 PL^Pdyn+^ neurons, but not excitatory inputs **(**Fig. 2**G-H)**. Addition of AMPA receptor antagonist NBQX (5 μM) and NMDA receptor antagonist CPP (5 μM) eliminated evoked inward excitatory postsynaptic currents **(**Fig. 2**G)** but not outward inhibitory postsynaptic currents **(**Fig. 2**H)**, which were could be blocked using the GABA-A receptor antagonist GABAzine (10 μM; Fig. 2**H)**. We did observe a reduction in outward inhibitory postsynaptic currents after treatment with NBQX and CPP **(**Fig. 2**H)**, which indicates that glutamatergic L2/3 PL^Pdyn+^ neurons can drive disynaptic feedforward inhibition of L5 PL neurons. These findings indicate that L2/3 PL^Pdyn+^ neurons consist of both glutamatergic and GABAergic phenotypes that have direct local connections onto L5 PL neurons.

### 3.2 The intrinsic firing of pyramidal L2 PL^Pdyn+^ neurons increases early after surgical incision

**Fig. 3:**
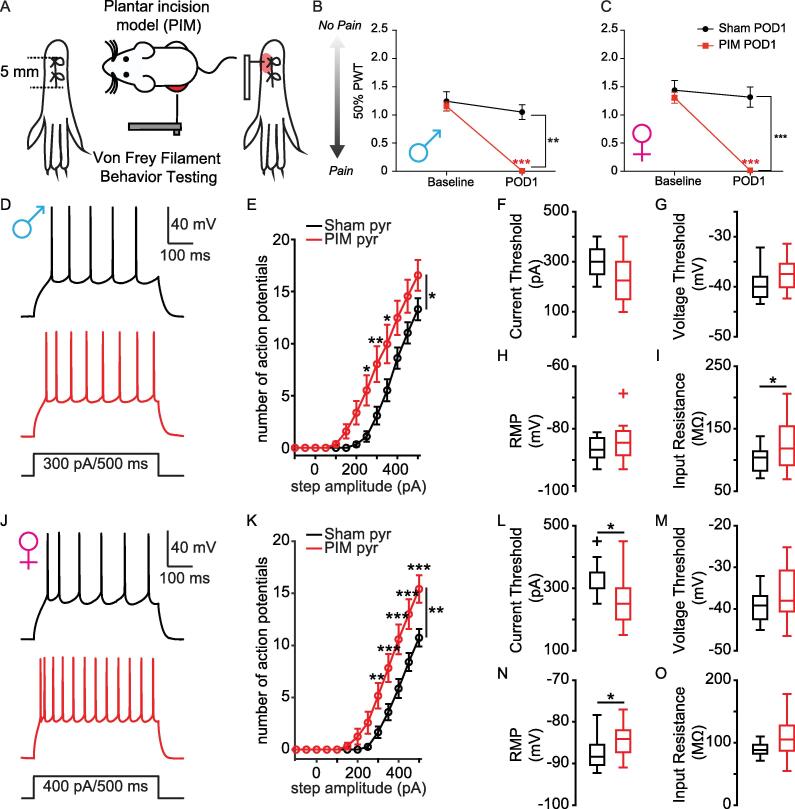
**The intrinsic firing of pyramidal L2 PL^Pdyn+^ neurons is increased early after surgical incision. A.** Schematic for plantar incision model of postoperative pain. **B**. Plantar incision induced mechanical allodynia in male mice (sham = 11; PIM = 10, ordinary-two-way ANOVA, F_surgery_ (1,19) = 14.41, P = ** 0.001, F_time_ (1, 19) = 77.16, *** P < 0.001, and F_interaction_ (1, 19) = 39.15, *** P < 0.001); Sidak’s post hoc, *** P < 0.001; Error bars = S.E.M.). **C**. Plantar incision induced mechanical allodynia in female mice (sham = 9, PIM = 10; ordinary-two-way ANOVA, F_surgery_ (1,17) = 26.08, *** P < 0.001, F_time_ (1, 17) = 37.81, *** P < 0.001, and F_interaction_ (1, 17) = 25.78, *** P < 0.001; Sidak’s post hoc, *** P < 0.001; Error bars = S.E.M.). **D.** Representative traces showing action potentials of pyramidal PL^Pdyn+^ neurons from sham POD1 (black) and PIM POD1 (red) male mice. **E.** The relationship of AP firing with current step amplitude from sham (n = 12 from 9 slices in 5 mice) and PIM (n = 14 from 8 slices in 5 mice) POD1 male mice (RM two-way ANOVA, F_surgery_ (1, 24) = 5.023, * P = 0.035, F_current_ (12,288) = 114.6, *** P < 0.001, and F_interaction_ (12,288) = 3.866, *** P < 0.001; Bonferroni post hoc, * P < 0.05, ** P < 0.01; Error bars = S.E.M.). **F-H.** Plantar incision did not alter (**F**) current threshold (t(24) = 1.4768, P = 0.15269), (**G**) voltage threshold, (t(24) = -1.466, P = 0.15564) or (**H**) resting membrane potential, (t(24) = -1.3605, P = 0.17948) of pyramidal L2 PL^Pdyn+^ neurons (Student’s *t*-test). Error bars = S.E.M.. **I.** Plantar incision significantly increased input resistance (t(24) = -2.0917, *P = 0.047228; Student’s *t*-test; Error bars = S.E.M.) of pyramidal L2 PL^Pdyn+^ neurons in male mice. **J.** representative traces showing action potentials of pyramidal PL^Pdyn+^ neurons from sham POD1 (black) and PIM POD1 (red) female mice. **K.** The relationship of AP firing with current step amplitude from sham (n = 15 from 10 slices in 6 mice) and PIM (n = 12 from 9 slices in 7 mice) POD1 female mice (RM two-way ANOVA, F_surgery_ (1,25) = 9.057, ** P = 0.006, F_current_ (12,300) = 141.7, *** P < 0.001, and F_interaction_ (12,300) = 7.217, *** P < 0.001; Bonferroni post hoc, **P < 0.01, ***P < 0.001; Error bars = S.E.M.). **L.** Plantar incision significantly decreased current threshold (t(25) = 2.5663, *P = 0.016654; Student’s *t*-test;;Error bars = S.E.M.). **M.** Plantar incision did not alter voltage threshold for AP firing (t(25) = -1.6148, P = 0.1190; Error bars = S.E.M.). **N**. Plantar incision significantly increased the resting membrane potential (t(25) = -2.1487, *P = 0.041538; Error bars = S.E.M.). **O**. Plantar incision did not alter input resistance (t(25) = -1.94, P = 0. 063734; Error bars = S.E.M.) of pyramidal L2 PL^Pdyn+^ neurons in female mice (Student’s *t*-test). (For interpretation of the references to colour in this figure legend, the reader is referred to the web version of this article.)

**Fig. 4:**
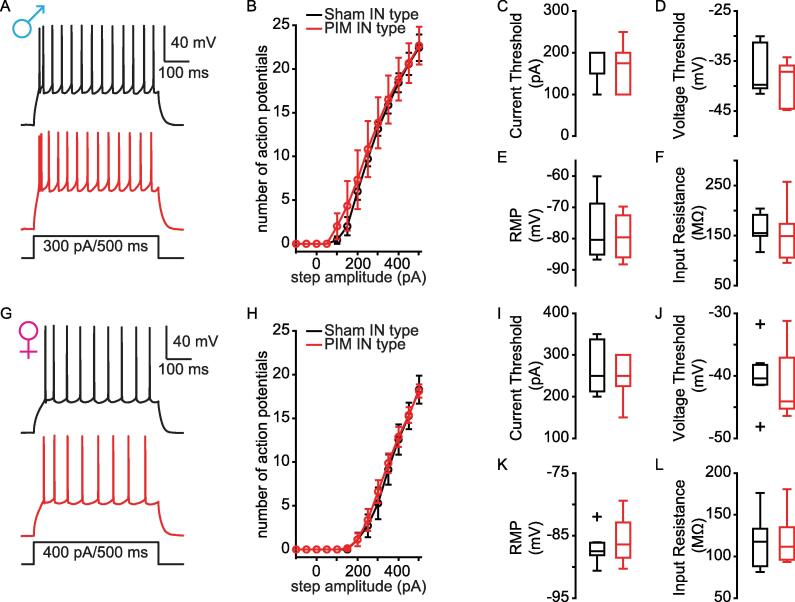
**Excitability of inhibitory L2 PL^Pdyn+^ neurons remained unchanged one day after plantar incision. A.** Representative traces showing action potentials of inhibitory PL^Pdyn+^ neurons from sham (black) and PIM (red) male mice. **B.** The relationship of AP firing with current step amplitude from sham (n = 8 from 4 slices in 3 mice) and PIM (n = 6 from 4 slices in 4 mice) POD1 male mice (RM two-way ANOVA, F_surgery_ (1,12) = 0.2866, P = 0.602, F_current_ (12,144) = 151.7, P < 0.001, F_interaction_ (12,144) = 0.3458, P = 0.979; Error bars = S.E.M.). **C-F.** Plantar incision produced no significant changes to (**C**) current threshold (Z(11) = 50, P = 1; Wilcoxon rank-sum test), (**D**) voltage threshold (t(11) = 0.86204, P = 0.40707; Student’s *t* test), (**E**) resting membrane potential (t(11) = 0.56753, P = 0.48175; Student’s *t*-test), or (**F**) input resistance (t(11) = 0.38175, P = 0.70991; Student’s *t*-test) of inhibitory L2 PL^Pdyn+^ neurons from male mice. Error bars = S.E.M.. **G.** Representative traces showing action potentials of inhibitory PL^Pdyn+^ neurons from sham (black) and PIM (red) female mice. **H.** The relationship of AP firing with current step amplitude from sham (n = 8 from 6 slices in 4 mice) and PIM (n = 8 from 5 slices in 4 mice) POD1 (RM two-way ANOVA, F_surgery_ (1,14) = 0.4498, P = 0.513; F_current_ (12,168) = 149.2, P < 0.001; F_interaction_ (12,168) = 0.4858, P = 0.921). **I-L.** Plantar incision produced no significant changes to (**I**) current threshold (t(14) = 1.0491, P = 0.3119; Students *t*-test), (**J**) voltage threshold (t(14) = 0.49749, P = 0.62656; Student’s *t*-test), (**K**) resting membrane potential (t(14) = -0.64363, P = 0.53022; Student’s *t*-test), or (**L**) input resistance (t(14) = 0.98769, P = 0.34007; Student’s *t*-test) of inhibitory L2 PL^Pdyn+^ neurons from female mice. Error bars = S.E.M.. (For interpretation of the references to colour in this figure legend, the reader is referred to the web version of this article.)

Our next goal was to determine whether surgical incision acutely alters the excitability L2 PL^Pdyn+^ neurons. For this, we applied the plantar incision model (PIM) of postoperative pain following previously established methods (Cowie and Stucky, 2019) **(**Fig. 3**A)**. We measured mechanical paw withdrawal threshold (PWT) prior to surgery (i.e. baseline) and changes to PWT were measured on postoperative day 1 (POD1). Both male and female mice in the PIM group displayed significant mechanical allodynia on POD1 compared to sham controls **(**Fig. 3**B, C)**. Following assessment of pain behavior on POD1, we prepared acute brain slices from PIM and sham mice for whole cell electrophysiological recordings of L2 PL^Pdyn+^ neurons. Analyses of recording data from the different neuronal subtypes (i.e. pyramidal and inhibitory) were based on the morphology and capacitance described in Fig. 1. Plantar incision significantly increased the excitability of pyramidal L2 PL^Pdyn+^ neurons in both male and female mice at POD1 **(**Fig. 3**D-O)**. This increase in excitability manifested as increased action potential (AP) firing in response to depolarizing current steps in both males **(**Fig. 3**D, E)** and females **(**Fig. 3**J, K)**. Further analysis revealed that plantar incision significantly increased input resistance of pyramidal L2 PL^Pdyn+^ neurons from male mice, but not did alter resting membrane potential nor current and voltage threshold for AP firing **(**Fig. 3**F-I)**. In female mice, plantar incision decreased the current threshold **(**Fig. 3**L,** Table 2**)** and depolarized the resting membrane potential **(**Fig. 3**O,** Table 2**)** for AP firing in pyramidal L2 PL^Pdyn+^ neurons without altering the voltage threshold **(**Fig. 3**M,** Table 2**)** or input resistance **(**Fig. 3**O,** Table 2**)**. These data suggest that the mechanisms by which surgical incision increass AP firing in pyramidal L2 PL^Pdyn+^ neurons differ between male and female mice. Interestingly, we found that plantar incision did not alter membrane nor excitable properties of inhibitory L2 PL^Pdyn+^ neurons at POD1 **(**Fig. 4**,** Table 3**)**.

**Table 2:** Comparison of pyramidal PL^Pdyn+^ neurons from sham and PIM POD1 male and female mice, related to Fig. 3.

**PL^Pdyn+^ neurons**	**Male**		**Female**	
	Sham (n = 12 neurons; 5 mice)	PIM (n = 14 neurons; 5 mice)	Sham (n = 15 neurons; 6 mice)	PIM (n = 12 neurons; 7 mice)
**Subthreshold properties**				
Resting potential (mV)	−86.3 ± 3.81	−83.6 ± 5.82	−87.7 ± 3.93	−84.3 ± 4.15^#*^
Input resistance (mΩ)	100 ± 20.42	126 ± 37.53^#*^	89.8 ± 3.1	109.5 ± 37.14
**Firing properties**				
Threshold (mV)	−39.6 ± 3.19	−37.6 ± 3.19	39.4 ± 3.51	−36.2 ± 6.58
Threshold (pA)	300 (1 0 0)^⊺^	225 (1 5 0)^⊺^	350 (50)^⊺^	250 (1 0 0)^⊺#*^
Frequency/current (Hz/pA)	0.1 ± 0.026	0.12 ± 0.021^$*^	0.1 ± 0.016	0.12 ± 0.026
APs @400 pA	8.5 ± 3.53	12.4 ± 6.16	8.4 ± 3.36	10.6 ± 4.94^#*^
Height (mV)	73.5 ± 8.8	71.7 ± 6.26	73 ± 9.03	68 ± 8.99

#: Student’s unpaired *t*-test; Data shown as mean ± standard deviation.

$: Mann-Whitney *U* test; Data shown are median ± standard deviation.

*: p < 0.05; **: p < 0.01; ***: p < 0.001.

⊺: median (interquartile range).

**Table 3:** Comparison of inhibitory PL^Pdyn+^ neurons from sham and PIM POD 1 male and female mice, related to Fig. 4.

**PL^Pdyn+^ neurons**	**Male**		**Female**	
	Sham (n = 8 neurons; 3 mice)	PIM (n = 6 neurons; 4 mice)	Sham (n = 8 neurons; 4 mice)	PIM (n = 8 neurons; 4 mice)
**Subthreshold properties**				
Resting potential (mV)	−77.8 ± 10.02 ^#*^	−79.3 ± 7.22	−86.7 ± 2.57	−85.7 ± 3.97
Input resistance (mΩ)	161 ± 29.19^#***^	155 ± 58.22	111 ± 11.8^#*^	120 ± 29.81
**Firing properties**				
Threshold (mV)	−36.8 ± 4.65	−39 ± 4.58	−40.1 ± 4.53	−41.3 ± 5.59
Threshold (pA)	200 (50)^⊺$***^	175 (1 0 0)^⊺^	275 (1 2 5)^⊺^	250 (75)^⊺^
Frequency/current (Hz/pA)	0.14 ± 0.022^$**^	0.16 ± 0.0678	0.13 ± 0.021^#***^	0.13 ± 0.0095
APs @400 pA	18.1 ± 2.8^#***^	18.8 ± 5.98	14.25 ± 4.74	12.9 ± 3.27
Height (mV)	60.6 ± 12.73^#*^	72 ± 11.61	74 ± 10.45	75.2 ± 16.3

#: Student’s unpaired *t*-test; Data shown as mean ± standard deviation.

$: Mann-Whitney *U* test; Data shown are median ± standard deviation.

*: p < 0.05; **: p < 0.01; ***: p < 0.001.

⊺: median (interquartile range).

### 3.3 Sex-specific changes to the excitability of L2 PL^Pdyn+^ neuronal subtypes are detected after the recovery from plantar incision

**Fig. 5:**
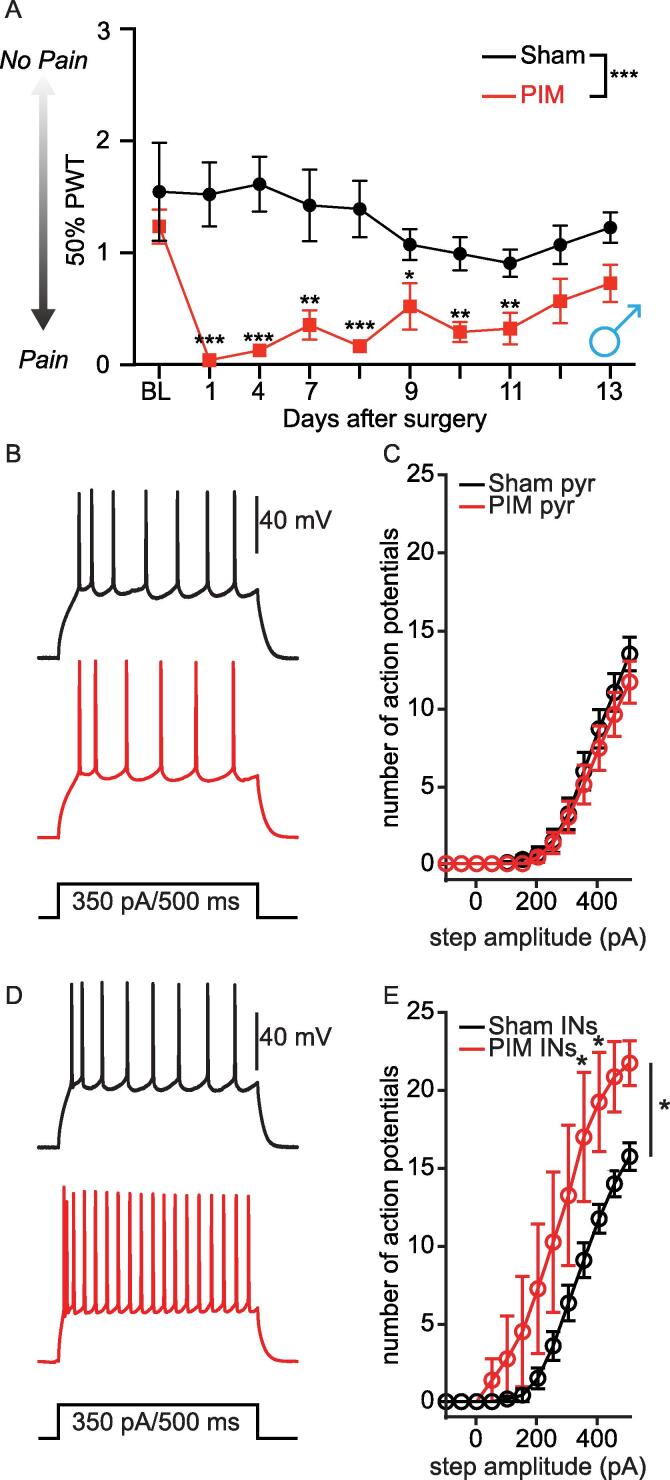
**Excitability of inhibitory L2 PL^Pdyn+^ neurons was increased when postoperative pain behavior subsided in male mice. A.** In mice with plantar incision, mean 50 % PTW gradually increased one day after PIM in male. (PIM = 5, sham = 5; RM two-way ANOVA, F_surgery_ (1,8) = 27.70, *** P < 0.001, F_time_ (9, 72) = 3.147, ** P = 0.003, and F_interaction_ (9, 72) = 3.186, ** P = 0.03); Dunnett post hoc, * P < 0.05 ** P < 0.01 *** P < 0.001; Error bars = S.E.M.). **B.** Representative traces showing action potentials of pyramidal PL^Pdyn+^ neurons from sham (black) and PIM (red) recovery male mice. **C.** The relationship of AP firing with current step amplitude of pyramidal L2 PL^Pdyn+^ neurons from sham (n = 15 from 7 slices in 5 mice) and PIM (n = 12 from 10 slices in 5 mice) recovery male mice (RM two-way ANOVA, F_surgery_ (1, 25) = 0.4025, P = 0.532, F_current_ (12,300) = 102.4, *** P < 0.001, F_interaction_ (12,300) = 0.5142, P = 0.905); Error bars = S.E.M.). **D.** Representative traces showing action potentials of inhibitory PL^Pdyn+^ neurons from sham (black) and PIM (red) recovery male mice. **E.** The relationship of AP firing with current step amplitude of inhibitory L2 PL^Pdyn+^ neurons from sham (n = 12 from 9 slices in 5 mice) and PIM (n = 8 from 6 slices in 5 mice) recovery male mice (RM two-way ANOVA, F_surgery_ (1, 18) = 4.756, * P = 0.043, F_current_ (12,216) = 71.79, *** P < 0.001, F_interaction_ (12,216) = 3.374, P < 0.001); Bonferroni post hoc, * P < 0.05; Error bars = S.E.M.). (For interpretation of the references to colour in this figure legend, the reader is referred to the web version of this article.)

**Fig. 6:**
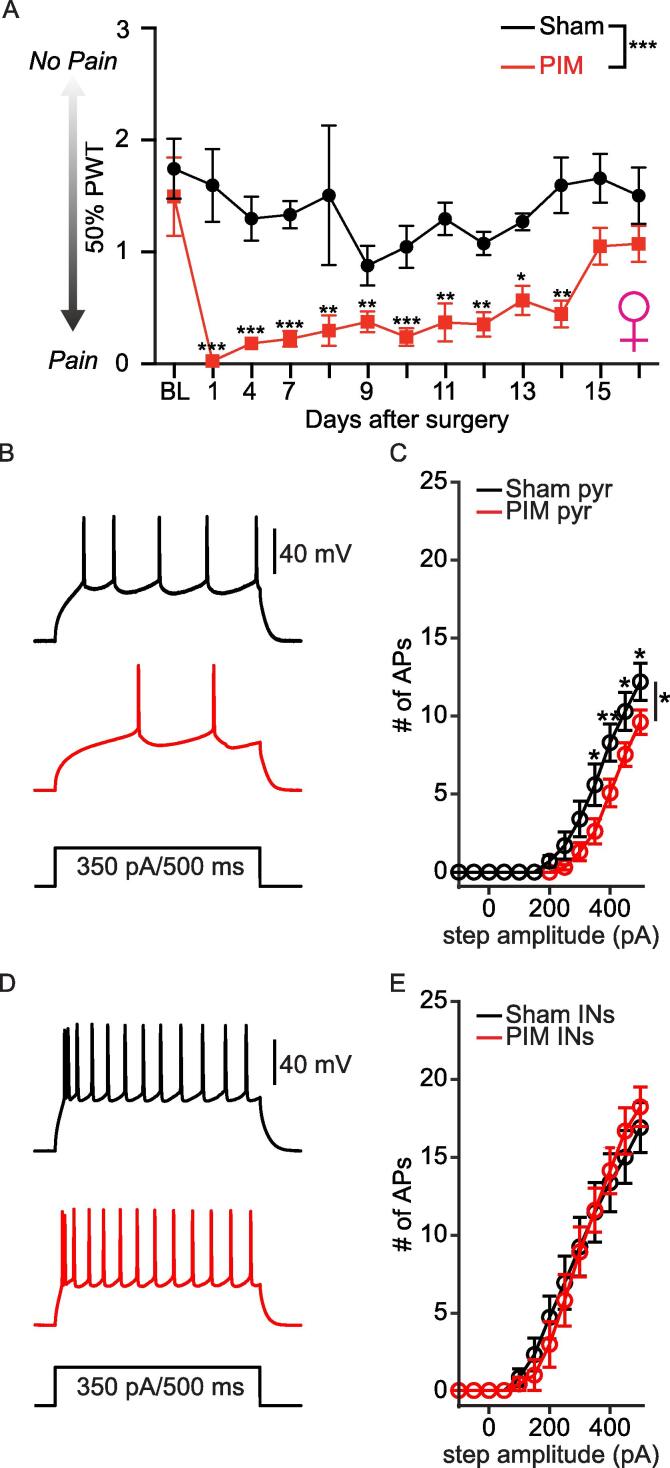
**Excitability of pyramidal PL^Pdyn+^ neurons was decreased when postoperative pain behavior subsided in female mice. A.** In mice with plantar incision, mean 50 % PTW gradually increased one day after PIM in female. (PIM = 5, sham = 5; RM two-way ANOVA, F_surgery_ (1,8) = 89.07, *** P < 0.001, F_time_ (12, 96) = 4.037, ** P < 0.01, and F_interaction_ (12, 96) = 1.514, P = 0.132; Dunnett post hoc, * P < 0.05, ** P < 0.01, *** P < 0.001; Error bars = S.E.M.). **B.** Representative traces showing action potentials of pyramidal PL^Pdyn+^ neurons from sham (black) and PIM (red) recovery female mice. **C.** The relationship of AP firing with current step amplitude of pyramidal L2 PL^Pdyn+^ neurons recorded from sham (n = 10 from 7 slices in 5 mice) and PIM (n = 13 from 8 slices in 5 mice) recovery female mice (RM two-way ANOVA, F_surgery_ (1, 21) = 4.333, * P = 0.05, F_current_ (12,252) = 121.1, *** P < 0.001, F_interaction_ (12,252) = 3.67, *** P < 0.001); Bonferroni post hoc, * P < 0.05, ** P < 0.01; Error bars = S.E.M.). **D.** Representative traces showing action potentials of inhibitory PL^Pdyn+^ neurons from sham (black) and PIM (red) recovery female mice. **E.** The relationship of AP firing with current step amplitude of inhibitory L2 PL^Pdyn+^ neurons from sham (n = 12 from 6 slices in 4 mice) and PIM (n = 7 from 6 slices in 5 mice) recovery female mice (RM two-way ANOVA, F_surgery_ (1, 17) = 0.002754, P = 0.959, F_current_ (12,204) = 102.3, *** P < 0.001, F_interaction_ (12,204) = 0.553, P = 0.877); Error bars = S.E.M.). (For interpretation of the references to colour in this figure legend, the reader is referred to the web version of this article.)

We next determined whether the initial hyperexcitation of pyramidal L2 PL^Pdyn+^ neurons was either attenuated or maintained following the recovery from plantar incision. For this, we removed sutures from the paw following behavior measurement at POD1 **(see Methods)**. Behavioral testing showed that PWT returned to baseline values approximately 2 weeks after plantar incision in male **(**Fig. 5**A)** and female **(**Fig. 6**A)** mice, which is consistent with previous studies (Pogatzki and Raja, 2003, Cowie and Stucky, 2019). Following the recovery, data analyses revealed no statistical differences in the excitable properties of pyramidal L2 PL^Pdyn+^ neurons between PIM and sham male mice **(**Fig. 5**B, C;** Table 4**)**. Furthermore, no significant difference was detected in the excitability of pyramidal PL^Pdyn+^ neurons from sham POD 1 and recovery mice. However, inhibitory L2 PL^Pdyn+^ neurons from male PIM mice displayed a significantly increased excitability following the recovery **(**Fig. 5**D, E;** Table 4**)**. Further analyses revealed that voltage threshold for AP firing in inhibitory L2 PL^Pdyn+^ neurons from male PIM recovery mice was significantly decreased, but no significant changes were detected in the current threshold, resting membrane potential or input resistance **(**Table 4**)**. Interestingly, pyramidal L2 PL^Pdyn+^ neurons recorded from the female PIM recovery group displayed a significant decrease in the excitability **(**Fig. 6**B, C)** with a depolarized RMP and decreased input resistance **(**Table 5**).** No statistical differences were detected in the inhibitory L2 PL^Pdyn+^ neurons compared to the female sham recovery group **(**Fig. 6**D, E;** Table 5**)**.

**Table 4:** Comparison of pyramidal and inhibitory PL^Pdyn+^ neurons from sham and PIM recovery male mice, related to Fig. 5.

**PL^Pdyn+^ neurons**	**PYR**		**INs**	
	Sham Recovery (n = 15 neurons; 5 mice)	PIM Recovery (n = 12 neurons; 5 mice)	Sham Recovery (n = 12 neurons; 5 mice)	PIM Recovery (n = 8 neurons; 5 mice)
**Subthreshold properties**				
Resting potential (mV)	−87.4 ± 5.96	−85.4 ± 2.97	−78.8 ± 5.77	−82.6 ± 7.8
Input resistance (mΩ)	95.8 ± 25.45	90.5 ± 19.17^$^	116.5 ± 21.74	128.7 ± 52.22
**Firing properties**				
Threshold (mV)	−42.6 ± 5.52	−40 ± 2.27^$^	−34.5 ± 6.96	−45.8 ± 6.93^#**^
Threshold (pA)	300 (1 0 0)^⊺^	300 (1 0 0)^⊺$^	250 (75)^⊺^	200 (1 2 5)^⊺^
Frequency/current (Hz/pA)	0.11 ± 0.021	0.11 ± 0.021	0.12 ± 0.01	0.19 ± 0.1^$**^
APs @400 pA	8.7 ± 4.76	8.4 ± 4.91	11.8 ± 3.22	19.25 ± 9
Height (mV)	75.1 ± 6.58	72 ± 6.65	67.9 ± 11.17	81.4 ± 13.78^$*^

#: Student’s unpaired *t*-test; Data shown as mean ± standard deviation.

$: Mann-Whitney *U* test; Data shown are median ± standard deviation.

*: p < 0.05; **: p < 0.01; ***: p < 0.001.

⊺: median (interquartile range).

**Table 5:** Comparison of pyramidal and inhibitory PL^Pdyn+^ neurons from sham and PIM recovery female mice, related to Fig. 6.

**PL^Pdyn+^ neurons**	**PYR**		**INs**	
	Sham Recovery (n = 10 neurons; 5 mice)	PIM Recovery (n = 13 neurons; 5 mice)	Sham Recovery (n = 12 neurons; 4 mice)	PIM Recovery (n = 7 neurons; 5 mice)
**Subthreshold properties**				
Resting potential (mV)	−83.9 ± 2.77	−80.9 ± 3.21^#*^	−79.2 ± 3.28	−77.1 ± 5.77
Input resistance (mΩ)	102.26 ± 19.08	86 ± 15.95^#*^	143.7 ± 41.36	137.3 ± 28.38
**Firing properties**				
Threshold (mV)	−35.7 ± 3.26	−35.7 ± 3.66	−33.8 ± 4.55	−35 ± 3.86
Threshold (pA)	300 (1 5 0)^⊺^	350 (1 2 5)^⊺^	200 (1 5 0)^⊺^	200 (50)^⊺^
Frequency/current (Hz/pA)	0.1 ± 0.017	0.098 ± 0.016	0.13 ± 0.031	0.12 ± 0.019
APs @400 pA	8.3 ± 3.77	5.1 ± 3.2^#*^	13.5 ± 1.9	14.3 ± 1.5
Height (mV)	73.4 ± 9.92	76.7 ± 9.35	66.6 ± 12.39	73.7 ± 12.59

#: Student’s unpaired *t*-test; Data shown as mean ± standard deviation.

$: Mann-Whitney *U* test; Data shown are median ± standard deviation.

*: p < 0.05; **: p < 0.01; ***: p < 0.001.

⊺: median (interquartile range).

### 3.4 Nerve injury induces a prolonged hyperexcitation of pyramidal L2 PL^Pdyn+^ neurons

**Fig. 7:**
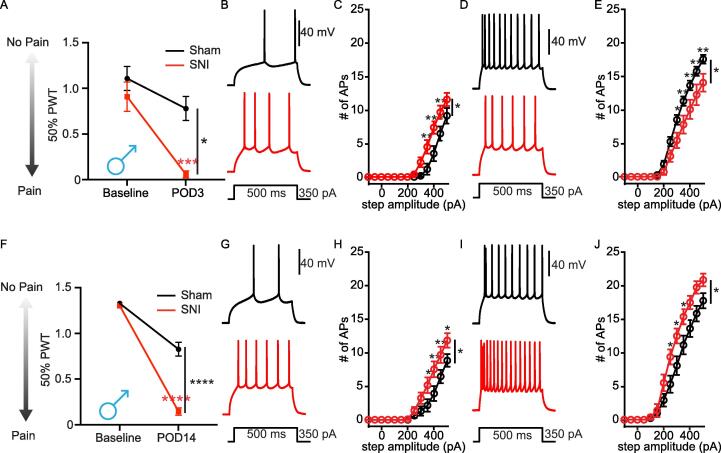
**Enhanced excitability of pyramidal L2 PL^Pdyn+^ neurons in the spared nerve injury (SNI) model of neuropathic pain in male mice. A.** SNI induced mechanical allodynia in male mice at POD3 (SNI = 5, sham = 5; ordinary-two-way ANOVA, F_surgery_ (1,8) = 10.38, * P = 0.0122, F_time_ (1, 8) = 33.46, *** P = 0.0004, and F_interaction_ (1, 8) = 6.538, * P = 0.0338; Sidak’s post hoc, *** P < 0.001; Error bars = S.E.M.). **B.** Representative traces showing action potentials of pyramidal PL^Pdyn+^ neurons from sham (black) and SNI (red) POD3 male mice. **C.** The relationship of AP firing with current step amplitude of pyramidal L2 PL^Pdyn+^ neurons from sham (n = 6 from 3 slices in 2 mice) and SNI (n = 10 from 8 slices in 4 mice) POD3 male mice (RM two-way ANOVA, F_surgery_ (1, 14) = 4.795, *P = 0.46, F_current_ (12,168) = 94.29, *** P < 0.001, F_interaction_ (12,168) = 4.472, *** P < 0.001); Bonferroni post hoc, ** P < 0.01 *** P < 0.001; Error bars = S.E.M.). **D.** Representative traces showing action potentials of inhibitory PL^Pdyn+^ neurons from sham (black) and SNI (red) POD3 male mice. **E.** The relationship of AP firing with current step amplitude of inhibitory L2 PL^Pdyn+^ neurons from sham (n = 15 from 7 slices in 4 mice) and SNI (n = 11 from 7 slices in 5 mice) POD3 male mice (RM two-way ANOVA, F_surgery_ (1, 24) = 6.175, * P = 0.020, F_current_ (12,288) = 263.8, *** P < 0.001, F_interaction_ (12,288) = 4.893, *** P < 0.001; Bonferroni post hoc, * P < 0.05, ** P < 0.01; Error bars = S.E.M.). **F.** SNI induced mechanical allodynia in male mice at POD14 (SNI = 5, sham = 5; ordinary-two-way ANOVA, F_surgery_ (1,8) = 57.87, *** P < 0.0001, F_time_ (1, 8) = 370.6, **** P < 0.0001, and F_interaction_ (1, 8) = 58.05, **** P < 0.0001; Sidak’s post hoc, **** P < 0.0001; Error bars = S.E.M.). **G.** Representative traces showing action potentials of pyramidal PL^Pdyn+^ neurons from sham (black) and SNI (red) POD14 male mice. **H.** The relationship of AP firing with current step amplitude of pyramidal L2 PL^Pdyn+^ neurons from sham (n = 13 from 9 slices in 5 mice) and SNI (n = 13 from 8 slices in 5 mice) POD14 male mice (RM two-way ANOVA, F_surgery_ (1, 24) = 4.334, * P = 0.048, F_current_ (12,288) = 4.334, *** P < 0.001, F_interaction_ (12,252) = 4.121, *** P < 0.001; Bonferroni post hoc, * P < 0.05, ** P < 0.01; Error bars = S.E.M.). **I.** Representative traces showing action potentials of inhibitory PL^Pdyn+^ neurons from sham (black) and SNI (red) POD14 male mice. **J.** The relationship of AP firing with current step amplitude of inhibitory L2 PL^Pdyn+^ neurons from sham (n = 13 from 7 slices in 5 mice) and SNI (n = 8 from 6 slices in 4 mice) POD14 male mice (Ordinary-two-way ANOVA, F_surgery_ (1, 19) = 4.46, * P = 0.048, F_current_ (12,228) = 234.8, *** P < 0.001, F_interaction_ (12,228) = 3.993, *** P < 0.001; Bonferroni post hoc, * P < 0.05; Error bars = S.E.M.). (For interpretation of the references to colour in this figure legend, the reader is referred to the web version of this article.)

We next applied the spared nerve injury (SNI) model of neuropathic pain, which allowed us to record L2 PL^Pdyn+^ neurons at early (POD3-4) and later (POD14-15) stages of pain development. Male mice displayed significant mechanical allodynia at both POD3 (Fig. 7**A**) and POD14 **(**Fig. 7**F)**. At POD3-4, the excitability of pyramidal L2 PL^Pdyn+^ neurons in male SNI was significantly enhanced **(**Fig. 7**B, C)** consistent with our findings from POD1 PIM male mice. Further analyses revealed a significant decrease in the current threshold for AP firing, but no difference in voltage threshold for AP firing, RMP, or input resistance **(**Table 6**)**. Conversely, the excitability of inhibitory L2 PL^Pdyn+^ neurons significantly decreased at the early stages of SNI **(**Fig. 7**D, E)**, including an increased current threshold for AP firing **(**Table 6**)**. At POD14-15, the excitability of pyramidal PL^Pdyn+^ neurons remained significantly increased in male SNI mice **(**Fig. 7**G, H,** Table 7**)**. However, in contrast to recordings from POD3-4, inhibitory PL^Pdyn+^ neurons recorded from POD14-15 SNI male mice displayed significantly more AP firing in response to depolarizing current steps together with an increased input resistance **(**Fig. 7**I, J,** Table 7**).** Further analyses showed that there was no statistical difference in the excitability of inhibitory PL^Pdyn+^ neurons from the sham group between POD3 and POD14 (POD3, n = 17, POD14, n = 17; Two-way ANOVA, F_time_ = 0.02851, P = 0.867; F_interaction_ = 0.2733, P = 0.993).

**Table 6:** Comparison of pyramidal and inhibitory PL^Pdyn+^ neurons from sham and SNI POD3 male mice, related to Fig. 7.


**PL^Pdyn+^ neurons**	**PYR**		**INs**	
	Sham (n = 6 neurons; 2 mice)	SNI (n = 10 neurons; 4 mice)	Sham (n = 15 neurons; 5 mice)	SNI (n = 11 neurons; 5 mice)
**Subthreshold properties**				
Resting potential (mV)	−80.7 ± 5.6	−79.4 ± 7.1	−82 ± 5.07	−77.6 ± 6.8
Input resistance (mΩ)	78.7 ± 9.06	88.6 ± 10.65	121.7 ± 16.82	112 ± 31.81
**Firing properties**				
Threshold (mV)	−36.1 ± 5.24	−35.7 ± 7.35	−37.4 ± 5.25	−33.5 ± 7.57
Threshold (pA)	400 (50)^⊺^	300 (1 0 0)^⊺#*^	200 (50)^⊺^	250 (1 0 0)^⊺$*^
Frequency/current (Hz/pA)	0.1 ± 0.018	0.1 ± 0.013	0.1 ± 0.016	0.1 ± 0.0097
APs @400 pA	3.5 ± 2.74	7.4 ± 2.95^#*^	13.7 ± 2.79	10.1 ± 4.72^#*^
Height (mV)	78.1 ± 4.67	79.7 ± 9.7	79.05 ± 8.77	71.9 ± 15.65

#: Student’s unpaired *t*-test; Data shown as mean ± standard deviation.

$: Mann-Whitney *U* test; Data shown are median ± standard deviation.

*: p < 0.05; **: p < 0.01; ***: p < 0.001.

⊺: median (interquartile range).

**Table 7:** Comparison of pyramidal and inhibitory PL^Pdyn+^ neurons from sham and SNI POD14 male mice, related to Fig. 7.

**PL^Pdyn+^ neurons**	**PYR**		**INs**	
	Sham (n = 13 neurons; 5 mice)	SNI (n = 13 neurons; 5 mice)	Sham (n = 13 neurons; 5 mice)	SNI (n = 8 neurons; 4 mice)
**Subthreshold properties**				
Resting potential (mV)	−79.8 ± 4.25	−82 ± 5.16	−77.8 ± 4.75	−79.4 ± 5.02
Input resistance (mΩ)	81.6 ± 22.05	94.3 ± 27.12	121.8 ± 34.75	153.2 ± 20.18^#*^
**Firing properties**				
Threshold (mV)	−34.7 ± 4.72	−37.4 ± 3.54	−36.7 ± 4.95	−35.1 ± 2.99
Threshold (pA)	400 (1 5 0)^⊺^	300 (1 2 5)^⊺^	200 (1 0 0)^⊺^	200 (50)^⊺^
Frequency/current (Hz/pA)	0.1 ± 0.019	0.1 ± 0.016	0.1 ± 0.018	0.1 ± 0.029
APs @400 pA	3.9 ± 4	7.5 ± 4.27^#*^	13.6 ± 4.52	19.4 ± 2.78^#^
Height (mV)	80.8 ± 7.91	76.6 ± 6.6	80.2 ± 9.92	75 ± 7.45

#: Student’s unpaired *t*-test; Data shown as mean ± standard deviation.

$: Mann-Whitney *U* test; Data shown are median ± standard deviation.

*: p < 0.05; **: p < 0.01; ***: p < 0.001.

⊺: median (interquartile range).

**Fig. 8:**
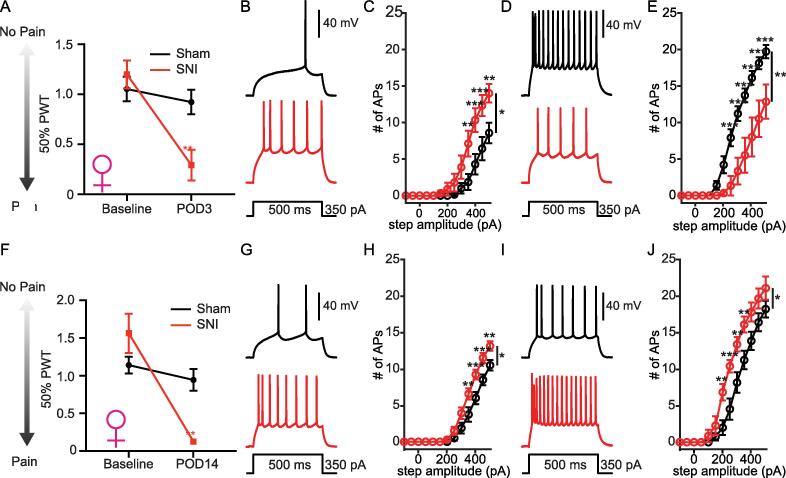
**Enhanced excitability of pyramidal L2 PL^Pdyn+^ neurons in the SNI female mice. A.** SNI induced mechanical allodynia in female mice at POD3 (SNI = 5, sham = 5; ordinary-two-way ANOVA, F_surgery_ (1,8) = 2.248, P = 0.1722, F_time_ (1, 8) = 22.09, P = 0.0015, and F_interaction_ (1, 8) = 12.39, P = 0.0078; Sidak’s post hoc, ** P < 0.01; Error bars = S.E.M.). **B.** Representative traces showing action potentials of pyramidal PL^Pdyn+^ neurons from sham (black) and SNI (red) POD3 female mice. **C.** The relationship of AP firing with current step amplitude of pyramidal L2 PL^Pdyn+^ neurons from sham (n = 7 from 5 slices in 4 mice) and SNI (n = 9 from 5 slices in 4 mice) POD3 female mice (RM two-way ANOVA, F_surgery_ (1, 14) = 5.605, * P = 0.033, F_current_ (12,168) = 55.79, *** P < 0.001, F_interaction_ (12,168) = 5.651, *** P < 0.001; Bonferroni post hoc, ** P < 0.01 *** P < 0.001; Error bars = S.E.M.). **D.** Representative traces showing action potentials of inhibitory PL^Pdyn+^ neurons from sham (black) and SNI (red) POD3 female mice. **E.** The relationship of AP firing with current step amplitude of inhibitory L2 PL^Pdyn+^ neurons from sham (n = 10 from 5 slices in 3 mice) and SNI (n = 6 from 3 slices in 3 mice) POD3 female mice (RM two-way ANOVA, F_surgery_ (1, 14) = 13.59, ** P = 0.002, F_current_ (12,168) = 115.3, *** P < 0.001, F_interaction_ (12,168) = 10.72, *** P < 0.001; Bonferroni post hoc, *** P < 0.001; Error bars = S.E.M.). **F.** SNI induced mechanical allodynia in female mice at POD14 (SNI = 6, sham = 6; ordinary-two-way ANOVA, F_surgery_ (1,10) = 0.007, P = 0.94, F_time_ (1, 10) = 16.77, ** P = 0.0022, and F_interaction_ (1, 10) = 10.99, ** P = 0.0078; Sidak’s post hoc, *** P < 0.001; Error bars = S.E.M.). **G.** Representative traces showing action potentials of pyramidal PL^Pdyn+^ neurons from sham (black) and SNI (red) POD14 female mice. **H.** The relationship of AP firing with current step amplitude of pyramidal L2 PL^Pdyn+^ neurons from sham (n = 8 from 6 slices in 5 mice) and SNI (n = 14 from 9 slices in 6 mice) POD14 female mice (RM two-way ANOVA, F_surgery_ (1, 20) = 5.86, * P = 0.025, F_current_ (12,240) = 214.7, *** P < 0.001, F_interaction_ (12,240) = 5.45, *** P < 0.001; Bonferroni post hoc, ** P < 0.01, *** P < 0.001; Error bars = S.E.M.). **I.** Representative traces showing action potentials of inhibitory PL^Pdyn+^ neurons from sham (black) and SNI (red) POD14 female mice. **J.** The relationship of AP firing with current step amplitude of inhibitory L2 PL^Pdyn+^ neurons from sham (n = 11 from 6 slices in 5 mice) and SNI (n = 7 from 4 slices in 3 mice) POD14 female mice (RM two-way ANOVA, F_surgery_ (1, 16) = 6.718, * P = 0.02, F_current_ (12,192) = 208.1, *** P < 0.001, F_interaction_ (12,192) = 4.579, *** P < 0.001; Bonferroni post hoc, ** P < 0.01, *** P < 0.001; Error bars = S.E.M.). (For interpretation of the references to colour in this figure legend, the reader is referred to the web version of this article.)

Female mice also displayed significant mechanical allodynia at both POD3 (Fig. 8**A**) and POD14 **(**Fig. 8**F)**. Similar to males, a significant increase and a decrease in the excitability of pyramidal and inhibitory L2 PL^Pdyn+^ neurons were measured in POD3-4 SNI female mice, respectively **(**Fig. 8**B-E)**. There was no statistical significance in the current or voltage threshold for AP firing, RMP, or input resistance in pyramidal PL^Pdyn+^ neurons **(**Table 8**)**. However, a significant increase in the current threshold for AP firing and a significant decrease in the input resistance was detected in inhibitory L2 PL^Pdyn+^ neurons 3 days after SNI **(**Table 8**)**. At POD14, the excitability of pyramidal L2 PL^Pdyn+^ neurons remained significantly increased in female SNI mice **(**Fig. 8**G, H)** with a decrease in the voltage threshold for AP firing **(**Table 9**).** In contrast to POD3-4, inhibitory L2 PL^Pdyn+^ neurons displayed significant hyperexcitability at POD14 **(**Fig. 8**I, J).** Further analyses revealed a decrease in both current and voltage threshold for AP firing and an increase in input resistance recorded from inhibitory L2 PL^Pdyn+^ neurons at POD14 **(**Table 9**)**.

**Table 8:** Comparison of pyramidal and inhibitory PL^Pdyn+^ neurons from sham and SNI POD3 female mice, related to Fig. 8.

**PL^Pdyn+^ neurons**	**PYR**		**INs**	
	Sham (n = 7 neurons; 4 mice)	SNI (n = 9 neurons; 4 mice)	Sham (n = 10 neurons; 3 mice)	SNI (n = 6 neurons; 3 mice)
**Subthreshold properties**				
Resting potential (mV)	−81 ± 5.21	−78.9 ± 3.96	−79.2 ± 4.12	−82.8 ± 2.39
Input resistance (mΩ)	82.1 ± 15.47	98.7 ± 23.4	131.2 ± 21.2	99.3 ± 22.7^#*^
**Firing properties**				
Threshold (mV)	−34.5 ± 5.78	−35.5 ± 4.69	−37 ± 3.95	−36.1 ± 3.33
Threshold (pA)	400 (1 5 0)^⊺^	300 (1 0 0)^⊺^	200 (1 0 0)^⊺^	300 (1 0 0)^⊺#**^
Frequency/current (Hz/pA)	0.1 ± 0.0098	0.12 ± 0.023	0.13 ± 0.021	0.12 ± 0.021
APs @400 pA	4.3 ± 3.73	10.3 ± 4.87^#*^	16.1 ± 3	8 ± 6.51
Height (mV)	79.2 ± 7.45	78.4 ± 9.17	79.9 ± 10.06	64.2 ± 13.44^#*^

#: Student’s unpaired *t*-test; Data shown as mean ± standard deviation.

$: Mann-Whitney *U* test; Data shown are median ± standard deviation.

*: p < 0.05; **: p < 0.01; ***: p < 0.001.

⊺: median (interquartile range).

**Table 9:** Comparison of pyramidal and inhibitory PL^Pdyn+^ neurons from sham and SNI POD14 female mice, related to Fig. 8.

**PL^Pdyn+^ neurons**	**PYR**		**INs**	
	Sham (n = 8 neurons; 5 mice)	SNI (n = 14 neurons; 6 mice)	Sham (n = 11 neurons; 5 mice)	SNI (n = 7 neurons; 3 mice)
**Subthreshold properties**				
Resting potential (mV)	−79.6 ± 5.75	−81.7 ± 3.95	−78.1 ± 4.33	−82.1 ± 6.75
Input resistance (mΩ)	91 ± 15.58	97.7 ± 15.76	121.4 ± 21.09	157.5 ± 38.95^#*^
**Firing properties**				
Threshold (mV)	−33 ± 5.67	−37.7 ± 3.64^#*^	−34.1 ± 4.76	−42.1 ± 4.65^#**^
Threshold (pA)	300 (1 0 0)^⊺^	300 (50)^⊺^	250 (1 0 0)^⊺^	200 (50)^⊺$*^
Frequency/current (Hz/pA)	0.09 ± 0.013	0.1 ± 0.014	0.13 ± 0.024	0.14 ± 0.022
APs @400 pA	6 ± 2.39	9.1 ± 2.49^#**^	13.9 ± 4.16	17.9 ± 3.44
Height (mV)	79.2 ± 5.1	80.5 ± 7.07	77.4 ± 12.84	76.3 ± 10.29

#: Student’s unpaired *t*-test; Data shown as mean ± standard deviation.

$: Mann-Whitney *U* test; Data shown are median ± standard deviation.

*: p < 0.05; **: p < 0.01; ***: p < 0.001.

⊺: median (interquartile range).

## 4 Discussion

In this study, we focused on characterizing the effects of surgical and neuropathic pain on the excitability of a subset of laminar layer 2 (L2) neurons in the prelimbic (PL) region of the mPFC that express prodynorphin (PL^Pdyn+^ neurons). Prodynorphin (Pdyn) is the precursor peptide for dynorphin (Dyn), the endogenous opioid ligand for the kappa opioid receptors (KOR), which are receptors involved in mediating sensory (Obara et al., 2003, Xu et al., 2004, Aita et al., 2010) and negative affective components of pain (Cahill et al., 2014, Massaly et al., 2019, Navratilova et al., 2019). We first present data showing that L2 PL^Pdyn+^ neurons are heterogeneous, consisting of both pyramidal (i.e. excitatory) and inhibitory neurons. We also show that L2/3 PL^Pdyn+^ neurons send both excitatory and inhibitory inputs to unlabeled L5 neurons in PL. This is consistent with the top-down organization of local circuits found in the mPFC and other cortical regions including motor and somatosensory cortex (Kampa et al., 2006, Weiler et al., 2008, Anderson et al., 2010, Hooks et al., 2011, Ferreira et al., 2015, Cheriyan and Sheets, 2018).

Electrophysiological recordings in acute brain slices revealed that the excitable properties of PL^Pdyn+^ neuronal subtypes are distinctly altered by surgical and nerve injury. One day after plantar incision (PI) of the hind paw, we identified a significant increase in the excitability of pyramidal, but not inhibitory, L2 PL^Pdyn+^ neurons in both male and female mice. Given that we demonstrate PL^Pdyn+^ neurons send projections locally within PL, this result suggest a potential increase in the local release of Dyn in PL soon after surgical injury (Abraham et al., 2021). The relevance of this finding is that KORs are expressed in mPFC (Mansour et al., 1988), and extensive work has shown that activation of KORs contribute to behaviors associated with negative affect such as aversion, fear, stress, and depression (McLaughlin et al., 2006, Knoll et al., 2007, Land et al., 2009, Ebner et al., 2010, Bruchas et al., 2011, Tejeda et al., 2013, Cahill et al., 2014). For example, activation of KORs produces conditioned place aversion (CPA) in rodents and dysphoria in humans (Bals-Kubik et al., 1993, Shippenberg et al., 1993, Knoll and Carlezon, 2010, Chavkin and Koob, 2016). Blocking KOR activity in the mPFC with KOR antagonist, nor-BNI, attenuates CPA induced by systemic administration of KOR agonist U69,593 (Tejeda et al., 2013). Together, this infers that hyperactivity of PL^Pdyn+^ neurons may contribute to an mPFC/KOR mediated aversion associated with acute surgical pain.

When pain behavior subsided approximately 2 weeks after PI, we observed no significant differences in the excitability of pyramidal L2 PL^Pdyn+^ neurons between sham and PIM male mice. This transient hyperexcitability of pyramidal L2 PL^Pdyn+^ neurons from PI supports the notion that the activity of these specific cells contribute to the expression and negative affect of postoperative pain that subsides following recovery from incision. However, we found a significant increase in the excitability of the inhibitory L2 PL^Pdyn+^ neurons in male mice following recovery from PI. One interpretation of these findings is that inhibitory L2 PL^Pdyn+^ neurons serve to dampen hyperexcitability in the PL during recovery from injury. However, the mechanisms that contribute to increased firing of inhibitory L2 PL^Pdyn+^ neurons and the overall effect of this change on PL circuits still need to be resolved. In the female mice recovered from PI, we observed a significant decrease in the excitability of pyramidal PL^Pdyn+^ neurons, but no difference in the inhibitory PL^Pdyn+^ neurons. This implies that the regulation of PL^Pdyn+^ neurons during recovery from surgical injury is different based on sex.

Previous work using a Pdyn knockout mouse indicated that Pdyn contributes to the maintenance but not the initiation of neuropathic pain (Wang et al., 2001). Interestingly, increased Pdyn mRNA levels are detected in the mPFC during the development of chronic pain (Candeletti and Ferri, 1995, Palmisano et al., 2018), but not in acute pain (Nwaneshiudu et al., 2019). We therefore questioned whether a prolonged increase in the excitability of the pyramidal PL^Pdyn+^ population would be detected both 3 days (initiation) and 2 weeks (maintenance) after spared nerve injury (SNI), a robust model of neuropathic pain. We found a significant increase in the excitability of pyramidal L2 PL^Pdyn+^ neurons in both male and female SNI mice both 3 days and 14 days after SNI. Interestingly, there was a dynamic change in the excitability of inhibitory L2 PL^Pdyn+^ neurons. Inhibitory PL^Pdyn+^ neurons were hypoexcitable 3 days after SNI but at 14 days after SNI we observed an increase in excitability of inhibitory PL^Pdyn+^ neurons compared to recordings from sham mice. Previous studies report that PL^Pdyn+^ inhibitory neurons express somatostatin (SOM) (Sohn et al., 2014, Loh et al., 2017, Smith et al., 2019). Our lab has reported that spontaneous excitatory synaptic inputs were reduced to L2/3 SOM expressing neurons of the PL in the female mice 7 days after SNI (Jones and Sheets, 2020), which is commonly considered an early time point in chronic neuropathic pain. Together with our current data, this indicates that reduced excitability of L2 Pdyn^+^/SOM^+^ GABAergic neurons in PL is a signature of the early stages of neuropathic pain. In addition, these findings suggest that increased Pdyn mRNA levels observed in the mPFC at later time points of neuropathic pain models (Candeletti and Ferri, 1995, Palmisano et al., 2018) result from prolonged hyperexcitability of both pyramidal and inhibitory PL^Pdyn+^ neurons.

A majority of PL^Pdyn+^ neurons identified in this study are located at L2/3 of PL, which is a laminar location that integrates a variety of excitatory inputs from regions such as the midline thalamus, contralateral mPFC, basolateral amygdala (BLA) and hippocampus (Hoover and Vertes, 2007, Little and Carter, 2012). Excitatory inputs from the BLA preferentially and monosynaptically target L2 corticoamygdalar (CA) neurons compared to neighboring neurons in the PL (Little and Carter, 2013). In the experiments where we injected AAV1-EF1a-DIO-hChR2(E123A)-EYFP into the PL cortex of Pdyn-Cre-tdTomato mice **(**Fig. 2**C)**, we found EYFP axonal labeling in the BLA (data not shown) indicating that L2 PL^Pdyn+^ neurons are CA neurons. These findings infer that L2 PL^Pdyn+^ neurons are targeted by the BLA. The relevance of a BLA → L2 PL^Pdyn+^ pathway is that hyperactivity of ascending BLA inputs to the mPFC occurs in arthritic and nerve-injury pain models (Ji et al., 2010, Zhang et al., 2015, Huang et al., 2019). Together, this suggests that the enhanced excitatory input from the BLA is contributing factor to increased excitability of L2 PL^Pdyn+^ neurons observed in the PIM and SNI models.

There are also indications for how pain-induced changes to PL^Pdyn+^ excitability affects local circuit activity within PL. Our lab has shown that L2/3 cortical neurons in the mPFC send local descending inputs onto L5 pyramidal neurons that project to the periaqueductal grey (PAG) (Cheriyan and Sheets, 2018). The PAG is a midbrain structure that plays a key role in endogenous analgesia (Reynolds, 1969). Reduced PL output to the PAG is implicated in neuropathic pain (Cheriyan and Sheets, 2018, Huang et al., 2019), and recent work reports that L2 CA activity driven by BLA inputs evokes strong local inhibition of L5 cortico-PAG neurons in the mPFC (Manoocheri and Carter, 2022). Based on these findings, our current working hypothesis to be tested in future studies is that incision or nerve injury activates and/or sensitizes the BLA → L2 PL^Pdyn+^ pathway enhancing local inhibition of L5 cortico-PAG neurons.

## 5 Conclusion

The mPFC play a significant role in regulating the sensory and affective components of pain. There is extensive evidence indicating functional changes within the mPFC in both acute and chronic pain modalities. However, published reports about how neurons with specific biomarker were regulated in both acute and chronic pain were scarce. In our study, we found different subtypes of prodynorphin expressing neurons in the mPFC and different subtypes exhibit distinct alterations in different pain modals and at different time point. The potential dynamic changes of different subtypes of Pdyn^+^ neurons may play a critical role in pain chronification. Moreover, Pdyn^+^ neurons produce dynorphin, which activate KORs. Studies have shown that activation of KORs in the mPFC regulates the affective aspect of pain. Thus, Pdyn^+^ neurons may serve as a potential target preventing pain chronification and attenuating affective aspects of pain.

## Author contributions

P.L.S. and S.Z. designed research and S.Z. performed all experiments. Y.Y. provided assistance with surgical procedures and behavioral analysis. S.Z. and P.L.S. analyzed data and wrote the paper.

## CRediT authorship contribution statement

**Shudi Zhou:** Conceptualization, Methodology, Validation, Formal analysis, Investigation, Writing – original draft, Writing – review & editing, Visualization, Project administration. **Yuexi Yin:** Investigation. **Patrick L. Sheets:** Conceptualization, Methodology, Validation, Formal analysis, Writing – original draft, Writing – review & editing, Visualization, Resources, Supervision, Project administration, Funding acquisition.

## Declaration of Competing Interest

The authors declare that they have no known competing financial interests or personal relationships that could have appeared to influence the work reported in this paper.

## Data Availability

Data will be made available on request.
